# Salvianolic Acid B Prevents Bone Loss in Prednisone-Treated Rats through Stimulation of Osteogenesis and Bone Marrow Angiogenesis

**DOI:** 10.1371/journal.pone.0034647

**Published:** 2012-04-06

**Authors:** Liao Cui, Ting Li, Yuyu Liu, Le Zhou, Pinghua Li, Bilian Xu, Lianfang Huang, Yan Chen, Yanzhi Liu, Xiaoyan Tian, Webster S. S. Jee, Tie Wu

**Affiliations:** 1 Department of Pharmacology, Guangdong Key Laboratory for Research and Development of Natural Drug, Guangdong Medical College, Zhanjiang City, Guangdong, China; 2 Division of Radiobiology, University of Utah School of Medicine, Salt Lake City, Utah, United States of America; Institut de Génomique Fonctionnelle de Lyon, France

## Abstract

Glucocorticoid (GC) induced osteoporosis (GIO) is caused by the long-term use of GC for treatment of autoimmune and inflammatory diseases. The GC related disruption of bone marrow microcirculation and increased adipogenesis contribute to GIO development. However, neither currently available anti-osteoporosis agent is completely addressed to microcirculation and bone marrow adipogenesis. Salvianolic acid B (Sal B) is a polyphenolic compound from a Chinese herbal medicine, *Salvia miltiorrhiza Bunge*. The aim of this study was to determine the effects of Sal B on osteoblast bone formation, angiogenesis and adipogenesis-associated GIO by performing marrow adipogenesis and microcirculation dilation and bone histomorphometry analyses. (1) *In vivo* study: Bone loss in GC treated rats was confirmed by significantly decreased BMD, bone strength, cancellous bone mass and architecture, osteoblast distribution, bone formation, marrow microvessel density and diameter along with down-regulation of marrow BMPs expression and increased adipogenesis. Daily treatment with Sal B (40 mg/kg/d) for 12 weeks in GC male rats prevented GC-induced cancellous bone loss and increased adipogenesis while increasing cancellous bone formation rate with improved local microcirculation by capillary dilation. Treatment with Sal B at a higher dose (80 mg/kg/d) not only prevented GC-induced osteopenia, but also increased cancellous bone mass and thickness, associated with increase of marrow BMPs expression, inhibited adipogenesis and further increased microvessel diameters. (2) *In vitro* study: In concentration from 10^−6^ mol/L to 10^−7^ mol/L, Sal B stimulated bone marrow stromal cell (MSC) differentiation to osteoblast and increased osteoblast activities, decreased GC associated adipogenic differentiation by down-regulation of PPARγ mRNA expression, increased Runx2 mRNA expression without osteoblast inducement, and, furthermore, Sal B decreased Dickkopf-1 and increased β-catenin mRNA expression with or without adipocyte inducement in MSC. We conclude that Sal B prevented bone loss in GC-treated rats through stimulation of osteogenesis, bone marrow angiogenesis and inhibition of adipogenesis.

## Introduction

Glucocorticoid (GC) therapy is commonly used for inflammatory and autoimmune diseases. The long-term administration of GC can lead to glucorcoticoid-induced osteoporosis (GIO), which significantly increases the patients' morbidity and mortality. Due to limited treatment options, the side effects of GC often have to be tolerated during treatment [1]. Currently, the clinical management of GIO relies on medications similar to those used for treatment of post-menopausal osteoporosis, such as calcium, vitamin D, bisphosphonates, raloxifene, PTH, hormone replacement and calcitonin. These drugs do not address the multi-factor driven GIO. In particular, they do not target the detrimental effect of GC on bone marrow fat metabolism and circulatory system [2]–[5]. Thus more studies on these GC induced effects may lead to development of a novel therapeutic approach to prevent and treat GIO.

The pathogenesis of GIO involves multiple factors, of which some suggest the decrease in number and functions of osteoblasts is the main contributing factor [2]. However, recently increased apoptosis of osteoblasts, osteocytes and endothelial cells, suppression of osteoblasts and osteoclasts, and endothelial cell precursor production as well as prolongation of the life span of osteoclasts have all been shown to contribute to the skeletal side effects of GC [4]–[6]. Recent studies suggested that the regulation of marrow stromal cell (MSC) differentiation into bone or fat cells [3] and the inhibition of bone marrow microvasculature play a very important role in GIO development [4], [5]. GC can inhibit osteoblast production of bone morphogenetic protein 2 (BMP-2), which causes decreased MSC differentiation into bone cells [7]. GC also directly induce differentiation of marrow stromal cells into adipocytes and inhibit osteogenic differentiation [8]. Kitajima et al. showed that mature fat cells exposed to high dose of GC were larger than control cells derived from bone marrow [9]. The latter would lead to narrowing and obstruction of capillaries in bone marrow microvasculature from increased adipose tissue that results in increased intraosseous pressure and decreased blood flows [5], [10]. Excessive GC treatment was also found to inhibit the growth of vascular endothelial cells that further contributes to microcirculation disturbance [11]. Marx et al. have previously demonstrated that the peroxisome proliferator-activated receptor γ (PPARγ) can induce apoptosis in vascular endothelial cells via caspase-3 activation, thus inhibiting vascular endothelial cell proliferation and angiogenesis [12]. GC can activate PPARγ in MSCs through different pathways to promote adipogenesis, which reduces osteoblast differentiation, and eventually leads to fat tissue accumulation in bone marrow [6]. Taken together, these studies suggest that the GIO bone loss is comprised of multiple mechanisms involving the increase in bone marrow adipogenesis and decrease in marrow angiogenesis leading to a decrease in bone marrow microvasculature and consequent decrease in osteogenesis [4], [5].

**Figure 1:**
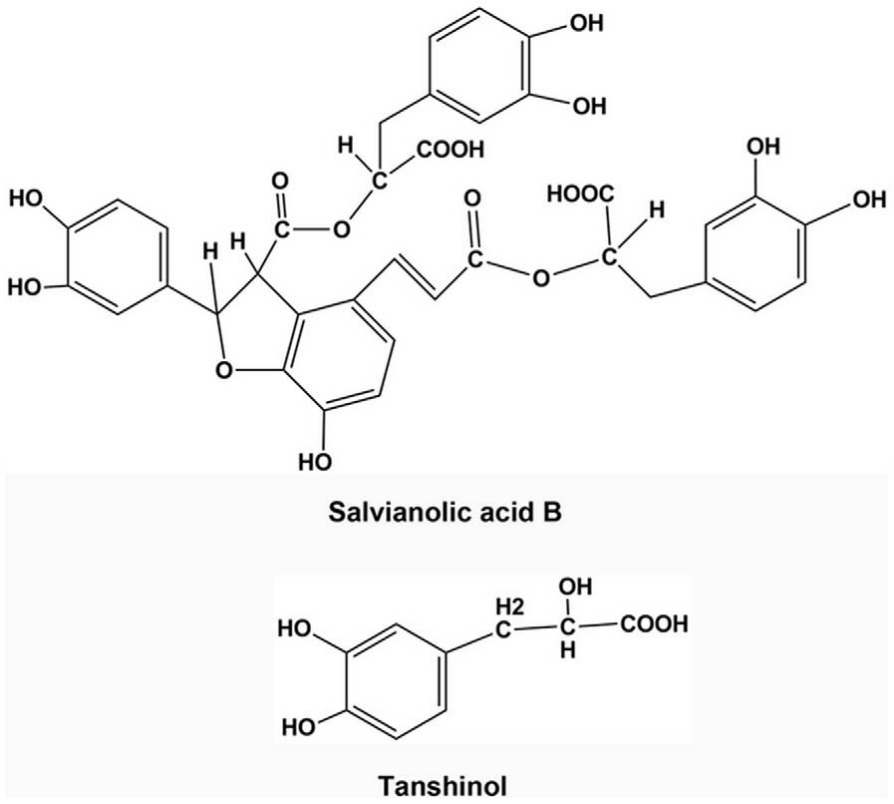
Structure of Salvianolic acid B and Tanshinol. Salvianolic acid B consists of three molecules of Tanshinol (D (+) β-3,4-dihydroxyphenyl lactic acid, danshensu) and a molecule of caffeic acid. Sal B can be converted *in vivo* to Tanshinol, another water-soluble bioactive ingredient of *Salvia miltiorrhiza Bunge*, with similar function to Sal B.

*Salvia miltiorrhiza Bunge* is a traditional Chinese medicine, called danshen, widely used in clinical practice for the prevention and treatment of cardio-cerebral vascular diseases. Pharmacological testing showed that danshen has anticoagulant, vasodilatory, increased blood flow, anti-inflammatory, free radical scavenging, mitochondrial protective and other activities [13]. Salvianolic acid B (Sal B), the aqueous bioactive component from *Salvia miltiorrhiza Bunge*, is a polyphenolic compound found in abundance in this plant. The structure of Sal B is shown in Figure 1 which consists of three molecules of Tanshinol, or called danshensu (D (+) β-3,4-dihydroxyphenyl lactic acid) and a molecule of caffeic acid, Sal B can be converted *in vivo* to Tanshinol (Salvianolic acid A, or Sal A), another water-soluble bioactive ingredient of *Salvia miltiorrhiza Bunge*, with similar function to Sal B. Both Sal B and Tanshinol are well-known as among the most effective natural product antioxidants [13], [14].

Multiple pharmacological studies have found that Sal B can attenuate the effect of myocardial ischemia-reperfusion injury [15]. Interestingly, Sal B can also increase angiogenesis and reduce myocardial ischemia via vascular endothelial growth factor (VEGF) activation [16]. It also relieves brain injury by reducing neuronal damages in cerebral ischemia [17]. Sal B can improve cellular hypoxia-ischemia by expanding micro-arteries, improving microcirculation and increasing the blood flow velocity. Its beneficial effects on blood vessel dilation and protection are believed to be mediated by blocking calcium channels and angiotensin-converting enzyme [18], [19]. Moreover, *Salvia miltiorrhiza Bunge* and its aqueous extract can increase the activity of superoxide dismutase, scavenge reactive oxygen species (ROS) and therefore reduce the damage of ROS to the vascular endothelium. Thus, Sal B functions as a vasodilator, maintains red blood cell deformability and increases the function of the hematopoietic system [20]. Recently, our *in vitro* studies have demonstrated that Sal A can inhibit glucocorticoid–induced bone marrow stromal cells adipogenesis, promote osteoblast differentiation, bone matrix formation and bone mineralization [21]. Therefore, we hypothesize that the clinical use of Sal B will hold promise for a more effective and safe treatment for GIO. The aim of the current study is to validate our hypothesis in a GIO rat model and additional study on in vitro.

## Materials and Methods

### Animals

Ethical Treatment of Animals: This study was carried out in strict accordance with the recommendations in the Guide for the Care and Use of Laboratory Animals of Guangdong Laboratory Animal Monitoring Institute under by National Laboratory Animal Monitoring Institute of China. The experiments have been conducted according to protocols approved for Specific Pathogen Free animal care of Animal Center of Guangdong Medical College, and approved by the Academic Committee on the Ethics of Animal Experiments of the Guangdong Medical College, Zhanjiang, P.R. China, Permit Number: SYXK(GUANGDONG) 2008-0007. All surgery was performed under sodium pentobarbital anesthesia.

The Sprague-Dawley male rats were acclimated to local vivarium conditions (temperature 24–26°C, humidity 67%) and allowed free access to water and diets containing 1.33% calcium, 0.95% phosphorus, and vitamin D3 60 IU %. All rats received subcutaneous injection of tetracycline (20 mg/kg, Sigma Chemical Co. St. Louis, MO) on days 13 and 14, and calcein (10 mg/kg, Sigma Chemical Co. St. Louis, MO) on 3 and 4 days before sacrifice.

### Experimental Protocols

Forty-six 6-month-old male Sprague-Dawley rats weighing 390±25 grams, were randomly divided into 6 groups with 8 rats per group, except 6 for the basal control group (Table 1). The groups were: 1) a basal (BAS), an age control (distilled water, CON), 3) 40 mg Sal B/kg/d (B40), 4) 3.5 mg prednisone acetate/kg/d (GC), 5) GC+40 mg Sal B/kg/d (GC+B40) and 6) GC+80 mg Sal B/kg/d (GC+B80). Prednisone acetate was obtained from Guangdong Xianju Pharmacy Co. China and the Sal B prepared as described in below. All treatments were by daily oral gavage for 12 weeks.

**Table 1:** Experimental Design.

Groups	Code	Description for Treatment and Dosage
(I) Basal control	BAS	Scarified at 0 day of the study
(II) Age control	CON	Vehicle treatment of distilled water at 5 ml/kg/d
(III) Intact+low dose Sal B	B40	Sal B treatment at 40 mg/kg/d
(IV) Prednisone model	GC	Prednisone acetate (Pred) at 3.5 mg/kg/d
(V) GC+low dose of Sal B	GC+B40	Pred at 3.5 mg/kg/d and Sal B at 40 mg/kg/d
(VI) GC+high dose of Sal B	GC+B80	Pred at 3.5 mg/kg/d and Sal B at 80 mg/kg/d

### Salvianolic acid B (Sal B) Preparation

**Figure 2:**
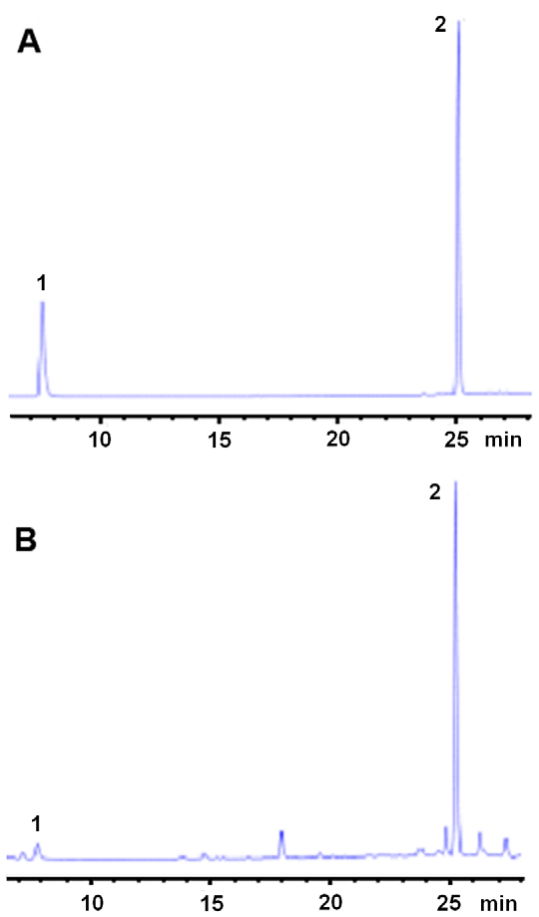
The HPLC analysis of control compounds (A) and aqueous extraction sample (B) showing the retention time of Tanshinol –Na (1) and Salvianolic acid B (2).

The original herbal medicine *Radix Salviae miltiorrhiza* was selected according to the standard protocol of the pharmacopoeia of the People's Republic of China [22]. The aqueous bioactive component from *Radix Salviae miltiorrhizae* was extracted as previously reported [23]. Salvianolic acid B (Sal B) and Tanshinol found in the aqueous extract of *Radix Salviae miltiorrhizae* were characterized by HPLC using a standard reference from the Chinese Biological Appraisal Institute, Beijing, China (>99.0%) [24]. HPLC chromatograms of the control compounds and aqueous extraction samples are shown in Figure 2. The content of Sal B was 25 mg per gram of *Radix Salviae miltiorrhiza*.

### Body weight and serum markers assay

Rats were weighed every week. At the end of the experiments, rats were sacrificed by cardiac puncture under anesthesia. Soft tissues were removed and weighed. Blood and serum samples were collected for measurements of serum calcium (Ca), the serum bone biomarkers alkaline phosphatase (ALP) and tartrate-resistant acid phosphatase-5b (TRACP-5b) according to manufacturers' instructions (Immunodiagnostic Systems Inc, USA).

### Immunohistochemical analysis of bone tissues

The distal femoral bone marrow cavity was exposed to prepare decalcified bone slides. Samples were decalcified at room temperature in 15% EDTA for 5 weeks. After decalcification, the sample was placed in 70% alcohol and paraffin embedded. Four micrometer paraffin slides were prepared on glass slides coated with egg white-glycerol, or polylysine, then dried for one hour at 60°C and stored at 4°C for future use.

Immunohistochemical analysis of bone marrow microcirculation factor VIII-related antigen (Von Willbrand Factor, vWF) and peroxisome proliferator-activated receptor γ (PPARγ) were performed following the manufacturers' instructions. Rabbit anti-human vWF polyclonal antibody was purchased from CHEMICON International, Inc, USA, and PPARγ mouse monoclonal antibody was purchased from Santa Cruz Biotechnology, Inc, USA. Briefly, the endogenous peroxidase activity of slides were blocked by 3% hydrogen peroxide and nonspecific binding was blocked with 10% goat serum. The slides were then incubated with primary antibody at 4°C overnight in a humidified chamber. The next day the slides were incubated with biotinylated secondary antibody for 15 min, washed again with PBS (2 min×3 times), then incubated with streptavidin conjugated to horseradish-peroxidase for 15 min. After a final PBS wash (5 min×3 times), a drop of freshly prepared DAB was applied on the slide for color development. The reaction was stopped when a uniform brown color became visible on the slide by rinsing in running water. Counterstaining was done with hematoxylin for 5–10 seconds. A control experiment was performed by replacing primary antibody with PBS. This method was a modification at the manufacturers' instructions.

The evaluation of Von Willbrand Factor expression and marrow microvessel density (MVD) were determined by counting the number of endothelial cells showing positive staining Von Willbrand Factor, and the MVD was determined by the total number of vWF stained cells divided by the measured area in previous studies [25], [26]. The diameter of marrow microvessels (DMV) was determined as the average diameters of microvessels in the same area used to measure MVD, using a digitizing image analysis system (Osteometrics, Inc. Decatur, GA, USA) To evaluate PPARγ positive expression in distal femur, the slides were scanned by LeicaQ550CW image analyzer and the average of absorbance from 30 positive expression cells on each slide was presented as mean ± SD (absorbance/30cells). Immunohistochemical analysis of bone marrow BMP-2 and BMP-7 expressions were performed following the manufacturers' instructions: Antigen retrieval was performed by high temperature and pressure treatment of the sections in citrate buffer (0.01 mol/L, pH 6.0). After a wash in PBS, sections were incubated with hydrogen peroxide blocking agent to quench endogenous tissue peroxidases and then washed in PBS. The slides were incubated with Ultra V Block for 30 min at room temperature. The primary antibodies were incubated according to the manufacturer's protocol (Maixin-Bio, China). Slides with serial sections were incubated with the primary antibodies (Santa Cluz Corporation) in a humidified chamber at 4°C overnight, followed by being incubated with streptavidin-perosidase avidin for 10 min at room temperature. Diaminobenzadine (DAB) staining was done by incubating the sections with coloring reagent. The sections were counterstained with Harris' haematoxylin, dehydrated through increasing concentrations of alcohol and mounted with coverslips. One section on each slide was used as a control to assess nonspecific binding. For this section, dilution buffer without antibody was used.

### Bone mineral density (BMD) determination

The right femurs of the rats were wrapped with saline saturated gauze to maintain moisture and stored at −20°C. After thawing at room temperature, the femurs were moisturized by soaking in saline solution, the residual muscle removed and the length of femur measured with a ruler. The femur bone mineral density between the midpoint and the distal end of the femur was scanned with a SD-1000 single-photon bone mineral density instrument (Nuclear Industry Beijing Institute of Geology) to measure the bone mineral content (BMC, g/cm) and bone width (BW, cm). The bone mineral density (BMD) measurements were performed at the midpoint of the femur and 2 cm proximal and the BMD was calculated as BMC/BW.

### Bone mechanical properties determination

After measuring BMD, the femur was used to determine the bone mechanical properties through three-point bending using Bose ElectroForce Testing system (ELF3510, Bose, USA). Bone samples were tested with a 1 mm indenter, at speed of 0.01 mm/s with a 15 mm span (L). Force (F) and deflection (D) that automatically recorded. The output parameters include elastic force (the force required to cause bone specimens to deform, N), maximum force (the maximum force the bone can resist, N), fracture force (the force required to cause bone fracture, N) and the maximum deflection (maximum degree of the bone displacement, mm). The stiffness coefficient (load-displacement curve slope, N/mm) was also calculated based on the above parameters.

### Bone Histomorphometry

The right proximal tibial growth plate and metaphyses were processed for cartilaginous longitudinal growth rate and cancellous bone histomorphometric analyses. The samples were opened to expose the bone marrow cavity using an Isomet Low Speed Saw (Buehler, Lake Bluff, Illinois, USA) and fixed in 10% phosphate buffered formalin for 24 hours. They were then dehydrated in graded ethanol, defatted in xylene, and embedded undecalcified in methyl methacrylate [27]. Frontal sections were cut at thicknesses of 4-and 10-µm. The 4-µm sections were stained by Goldner's Trichrome for static histomorphometric measurements. The 10-µm unstained sections were used for dynamic histomorphometric analyses [28].

A digitizing image analysis system (Osteometrics, Inc. Decatur, GA, USA) was used for quantitative bone histomorphometric measurements. Briefly, the regional of interest were the proximal tibial growth plate and the proximal tibial metaphysis (PTM) located between 1 and 4 mm distal to the growth plate-epiphyseal junction. Static measurements included total tissue volume (TV), trabecular bone volume (BV), marrow fatty area (F.Ar), trabecular bone surface (BS), osteoclast surface (OcS) and osteoblast surface (ObS). Dynamic measurements include interlabel width in the growth plate (G-Int.Wi) of PTM, trabecular single-labeled surface (sL.S), double labeled surface (dL.S) and interlabel width (Int.Wi), and endocortical single-labeled surface (Ec-sL.S), double labeled surface (Ec-dL.S) and endocortical interlabel width (Ec-Int.Wi). These parameters were used to calculate longitudinal growth rate (LGR), percentages of trabecular bone volume (BV/TV), trabecular number (Tb.N), trabecular thickness (Tb.Th), trabecular separation (Tb.Sp), marrow fatty area (F.Ar/TV), osteoclast surface (OcS/BS), osteoblast surface (ObS/BS), longitudinal growth rate (LGR), mineralizing surface (MS/BS), mineral apposition rate (MAR), bone formation rate (BFR) per unit of bone surface (BFR/BS), bone volume (BFR/BV), and tissue volume (BFR/TV), endocortical mineralizing surface (Ec-MS/BS), endocortical mineral apposition rate (Ec-MAR), endocortical bone formation rate (Ec-BFR) per unit of bone surface (Ec-BFR/BS) as previously described [29], [30].

### Culture of rat osteoblast and marrow stromal cell (MSC)

Osteoblastic cell was isolated from newborn rat calvaria (rOB). After removing the periosteum, the bones were cut into small flaps of 1 mm^3^ and subjected to digestion with 0.25% trypsin (Life Technologies Gibco-BRL) for 20 min at 37 C. After centrifugation, supernatants were discarded to remove the fibroblast population. Then the flaps were digestion with 0.2% collagenase type I and 0.1% hyaluronidase (Life Technologies Gibco-BRL) for six 20 min intervals at 37 C. The samples were then washed thoroughly with Dulbecco's modified eagle's medium (DMEM, Gibco). The precipitates and bone flaps were transferred to 25 cm^2^ culture flasks and cultured in Dulbecco's modified eagle's medium, 100 U/mL penicillin, 100 µg/mL streptomycin and 10% fetal bovine serum (FBS, Gibco) at 37°C in a humidified incubator with 5% carbon dioxide (CO_2_). The DMEM culture medium was changed every 3 days. MTT (3-(4,5-Dimethylthiazol-2-yl)-2,5-diphenyltetrazolium bromide) (Bio-Rad, USA) assay was use for the observation of cell growth/death.

Primary rat bone marrow stromal cells (rMSC) were collected from marrow of femur in 4-week-old Wistar rats (obtained from the Laboratory Animal Center of Guangdong Medical College) [21]. The rMSC then were prepared by gradient centrifugation at 900×*g* for 30 min on a Percoll-Paque gradient (Amersham Pharmacia, USA) at a specific gravity of 1.073 g/mL. The low-density mononuclear cells were washed twice in Hanks' balanced salt soltion and cultured in DMEM supplemented with 10% FBS and 1% antibiotic (10 000 U/mL penicillin G sodium, 10000 µg/mL streptomycin sulfate) at 37 C in a humid atmosphere containing 5% CO. The medium was changed every 3 to 4 d to remove the non-adherent hematopoietic cells. The adherent cell population was expanded and passaged every 12 to 14 d. All cells used for the experiments have been through three passages. To identify the abilities of rMSC with regard to osteogenesis and adipogenesis, the following studies were performed.

Induction of osteogenic differentiation of rMSC (OB-in): When the rMSC from passage number 2 became 80% confluent in the culture plates, the culture medium was changed to an osteoblast inducing medium containing 50 µg/mL L-ascorbic acid, 10^−2^ mol/L β-glycerophosphate, and 10^−8^ mol/L dexamethasone. When the cells became layered and confluent up to 100%, visible symmetric colonies were observed after osteoblast induction.

Induction of adipogenic differentiation of rMSC (Ad-in): The rMSC of each group were replated at 2×104 cells/cm^2^ in a 25 cm^2^ culture dish and maintained until 80% confluence. Then, the culture medium was switched to adipogenic medium consisting of control medium supplemented with 10 µg/ml insulin (Sigma, St. Louis, MO), 10^−5^ mol/L dexamethasone (Sigma, St. Louis, MO), 2×10^−4^ mol/L indomethacin (Sigma, St. Louis, MO) and 5×10^−5^ mol/L isobutylmethyl xanthine (IBMX, Sigma, St. Louis, MO) for an additional 5 days followed by total RNA isolation.

Identification for OB-in and Ad-in of MSC had according to the methods referred to Cui. et al. [21].

### Determination of alkaline phosphatase (ALP) and osteocalcin secretion

ALP activity assay: Cells were seeded in 96-well plates and the confluent cells were cultured for the indicated period with or without Sal B treatment. Cells were washed with PBS and 150 µL of substrat buffer (6.7 mmol/L disodium p-nitrophenylphosphate hexahydrate, 25 mmol/L diethanolamine and 1 mmol/L MgCl) was added. After the mixtures were incubated at 37°C for 3 min, we measured the absorbance at 405 nm.

Determination of osteocalcin secretion: Cells were seeded in 6-well plates at a seeding density of 2×10^4^cells/cm^2^ and were further cultured with or without Sal B treatment for 14 d. At the end of the culture, the conditioned media were collected for assessment. The concentration of free osteocalcin was measured by radioimmunoassay (RIA) according to the manufacturer's instructions (Tian Jin Nine Tripods Medical& Bioengineering Co, LTD). The intra-assay variance of the measurements of osteocalcin RIA was 1.26%.

### Reverse transcription polymerase chain reaction (RT-PCR) assay

Test of cultured cells: Total RNA was extracted from cultured cells using TRIZOL reagent (Invitrogen, Carlsbad, CA) and subjected to RT-PCR analysis using the 9600Gen Amp PCR system (PerkinElmer Applied Biosystems, USA) with PCR reagents (Invitrogen, Carlsbad, CA) according to the manufacturer's protocol. Primers used are listed in Table 2. Amplified products were then loaded on a 2% agarose gel and subjected to electrophoresis. Digital pictures were taken and analyzed with gel image system. Each gene expression was normalized to GAPDH.

**Table 2:** Primer sequences used in RT-PCR.

Gene	Primer sequence		Products Length (bp)
Runx2	5′-ACTGAAGAGGCTGTTTGACG-3′	(sense)	122
	5′-TCACTACCAGCCACCGAGA-3′	(antisense)	
PPARγ	5′-GCCTTGCTGTGGGGATGTCT-3′	(sense)	240
	5′-CGAAACTGGCACCCTTGAAAAAT-3′	(antisense)	
Dickkopf-1	5′-GCCTCCGATCATCAGACGGT-3′	(sense)	224
	5′-GCAGGTGTGGAGCCTAGAAG-3′	(antisense)	
β-catenin	5′-TGCAGCGACTAAGCAGGA-3′	(sense)	198
	5′-TCACCAGCACGAAGGACA-3′	(antisense)	
GAPDH	5′-ACCACAGTCCATGCCATCAC-3′	(sense)	451
	5′-TCCACCACCCTGTTGCTGTA-3′	(antisense)	

Test of rat femur bone tissue: The upper femur halves obtained were snap-frozen in liquid nitrogen and stored at −70°C. Total RNA was prepared using TRIZOL reagent and stored at −20°C in ribonuclease (RNase)-free water until use. To determine mRNA levels, a one-step reverse transcription-polymerase chain reaction (RT-PCR) procedure was performed using the 9600Gen Amp PCR system (PerkinElmer Applied Biosystems, USA) according to the manufacturer's protocol. Primers used are listed in Table 3. Amplified products were then loaded on a 2% agarose gel and subjected to electrophoresis. Digital pictures were taken and analyzed using Bandscan analysis software. Each gene expression was normalized to GAPDH.

**Table 3:** Primer sequences used in RT-PCR.

Gene	Primer sequence		Products Length (bp)
BMP-2	5′-AAATTATAAAGCCTGCCACAG-3′	(sense)	326
	5′-TTGACGCTTTTCTCGTTTGTG-3′	(antisense)	
BMP-7	5′-AGACGCCAAAGAACCAAGAG-3′	(sense)	323
	5′-GCTGTCGTCGAAGTAGAGGA-3′	(antisense)	
GAPDH	5′-CCATGGAGAAGGCTGGGG-3′	(sense)	195
	5′-CAAAGTTGTCATGGATGACC-3′	(antisense)	

### Statistic Analysis

Data were presented as mean± SD. The statistical differences among groups were evaluated using variance (ANOVA) with Fisher's PLSD test. Probabilities (*p*) less than 0.05 were considered significant.

## Results

### Effect of Aging

**Figure 3:**
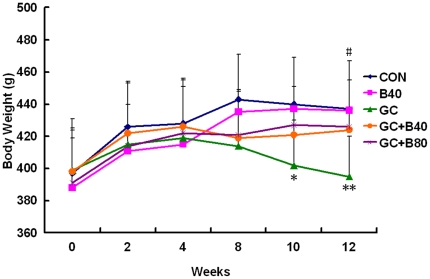
Body weight (g) changes during the experimental period. Body weight measurements from vehicle (aging) control (CON), Sal B40 mg/kg/d alone (B40), prednisone alone (GC), GC plus 40 mg Sal B/kg/d (GC+B40) and GC plus 80 mg Sal B/kg/d (GC+B80) treated rats. Only the GC treated rats showed a significant 10% lower body weight versus CON. Significant weight loss began at 10 weeks post treatment. *P<0.05, ** *P*<0.01 *vs* CON; ^#^ *P*<0.05, ^##^ *P*<0.01 *vs* GC.

Aging has a significant impact on the bone architecture and microvasculature density. Compared with the 6 month old rat, the aging associated changes seen at 9 months included significantly increased femoral cortical bone strength parameters (i.e. the maximum, fracture and elastic forces increased by 28, 33 and 34%), decreased proximal tibial (PT) longitudinal growth rate (LGR) (down by 38%), decreased proximal tibial metaphyses (PTM) cancellous bone formation (i.e. MS/BS, BFR/BS, BFR/BV, BFR/TV down by 25, 28, 26 and 44%) and endocortical bone formation indices (i.e. Ec-MS/BS, Ec-MAR and Ec-BFR/BS by 26, 49 and 62%), these changes were associated with significantly increased distal femoral marrow microvessels diameter (DMV), up by 54%. No available data of soft tissue in 6 month old rat. (Tables 4–9, Figures 3–9).

**Figure 4 pone-0034647-g004:**
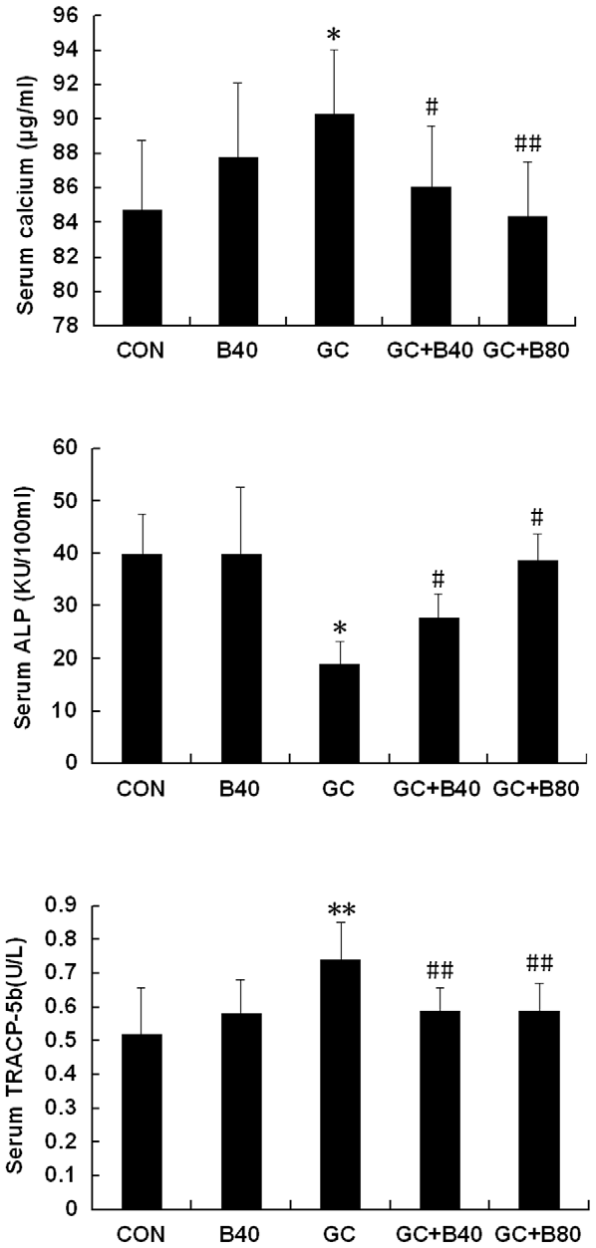
Biomarker changes in the blood. Serum calcium (Ca, µg/ml), alkaline phosphatase (ALP, KU/100 ml) and tartrate-resistant acid phosphatase-5b (TRACP-5b, U/L) from vehicle (aging) control (CON), Sal B40 mg/kg/d alone (B40), prednisone alone (GC), GC plus 40 mg Sal B/kg/d (GC+B40) and GC plus 80 mg Sal B/kg/d (GC+B80) treated rats. The GC treatment significantly increased serum calcium and TRACP-5b and reduced ALP, while the Sal B significantly inhibited these GC induced changes. Value are mean ± SD, *P<0.05, ** *P*<0.01 *vs* CON; ^#^ *P*<0.05, ^# #^ *P*<0.01 *vs* GC.

**Figure 5 pone-0034647-g005:**
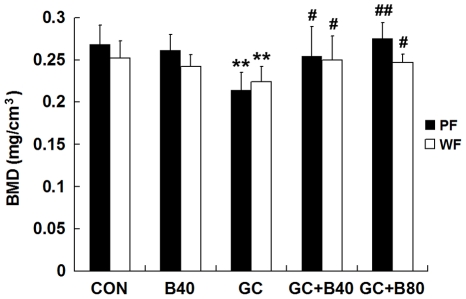
Effects of different groups on Bone Mineral Density (BMD, mg/cm). BMD measurements of proximal femur (PF) and whole femur (WF) from vehicle (aging) control (CON), Sal B40 mg/kg/d alone (B40), prednisone alone (GC), GC plus 40 mg Sal B/kg/d (GC+B40) and GC plus 80 mg Sal B/kg/d (GC+B80) treated rats. GC treatment significantly reduced BMD at both sites, while Sal B prevented this reduction in GC treated rats. Value are mean ± SD, *P<0.05, ** *P*<0.01 *vs* CON; ^#^ *P*<0.05, ^# #^ *P*<0.01 *vs* GC.

**Figure 6 pone-0034647-g006:**
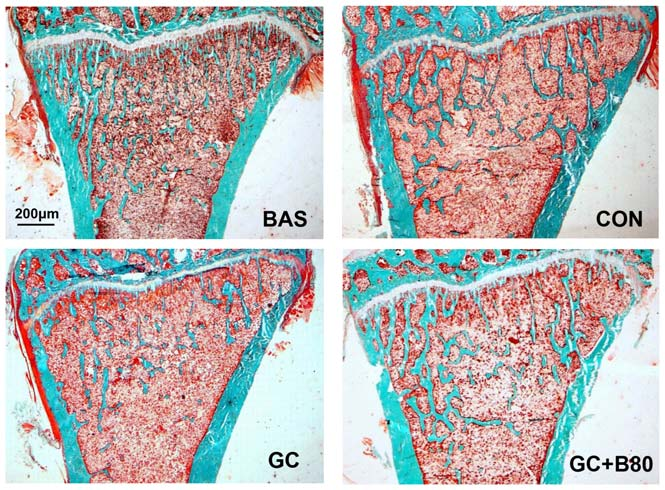
Effects of different treatments on proximal tibial metaphyses (PTM) bone structure and trabecular mass. Representative micrographs of PTM from basal (BAS), vehicle (aging) control (CON), prednisone (GC) and GC plus 80 mg Sal B/kg/d (GC+B80) treated rats. BAS: 6-month-old beginning control. CON: 9-month-old terminal vehicle control with fewer trabeculae. GC: Prednisone induced further reduction in cancellous bone mass and thinner trabeculae versus CON. GC+B80: Sal B80 treated GC rats had increased cancellous bone mass with thicker trabeculae compared to BAS and CON. (Masson-Goldner Trichrome stain: trabecular in green stain). Quantitative measurements of static parameters are shown in table 6.

**Figure 7 pone-0034647-g007:**
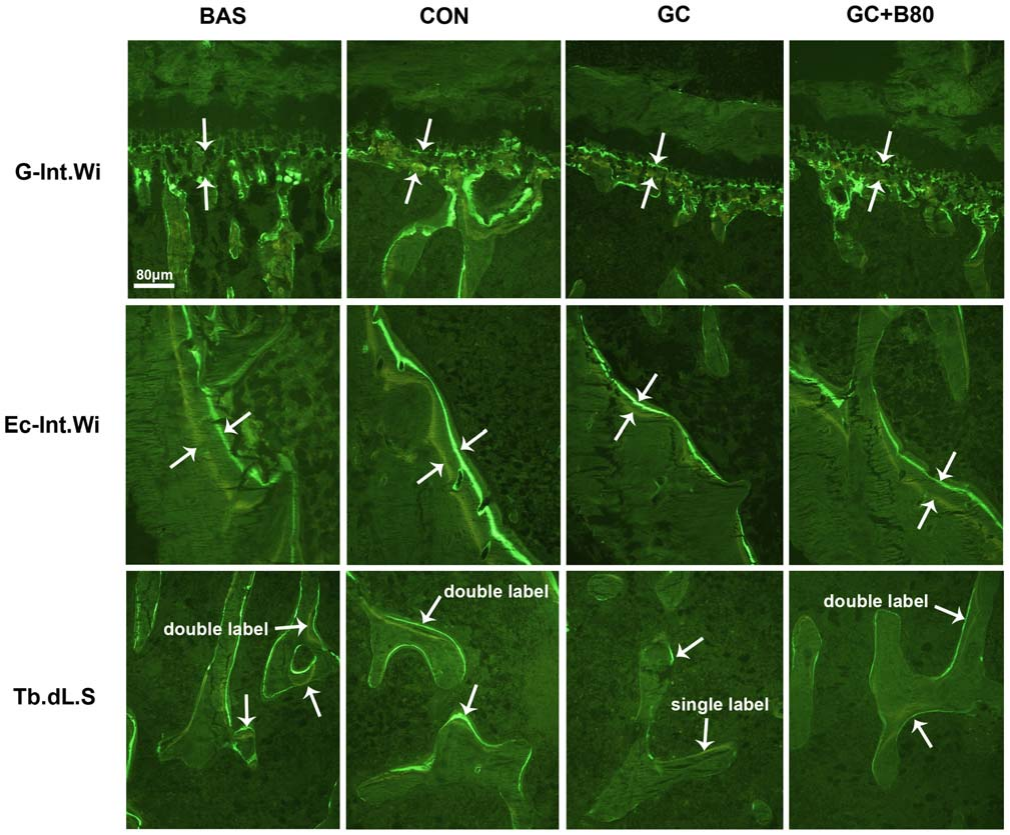
Effects of different treatments on Proximal tibial cartilage growth and mineral bone formation. Representative fluorescence micrographs of interlabel width in the growth plate (G-Int.Wi), interlabel width in the endocortical (Ec-Int.Wi) and double labeling in trabecular surface (Tb.dL.S) in basal (BAS), vehicle (aging) control (CON), prednisone alone (GC) and GC plus 80 mg Sal B/kg/d (GC+B80) treated rats. Arrows point to interlabeling distances after double labeling with tetracycline and calcein. The interlabeling distance in the growth plate was used to determine longitudinal growth rate (LGR). There were age-related decreases in LGR and in endocortical bone formation from 6 month (BAS) and 9 month (CON) and a further reduction with prednisone alone treatment (GC), while 80 mg/kg/d of Sal B (GC+80) prevented the GC-induced reduction. There was an absence of double labeling in the trabecular surface after GC treatment, while the GC+B80 section exhibited similar double labeling to BAS and CON rats. (Villanueva bone stain under fluorescence light). Quantitative measurements of dynamic parameters are shown in table 7.

**Figure 8 pone-0034647-g008:**
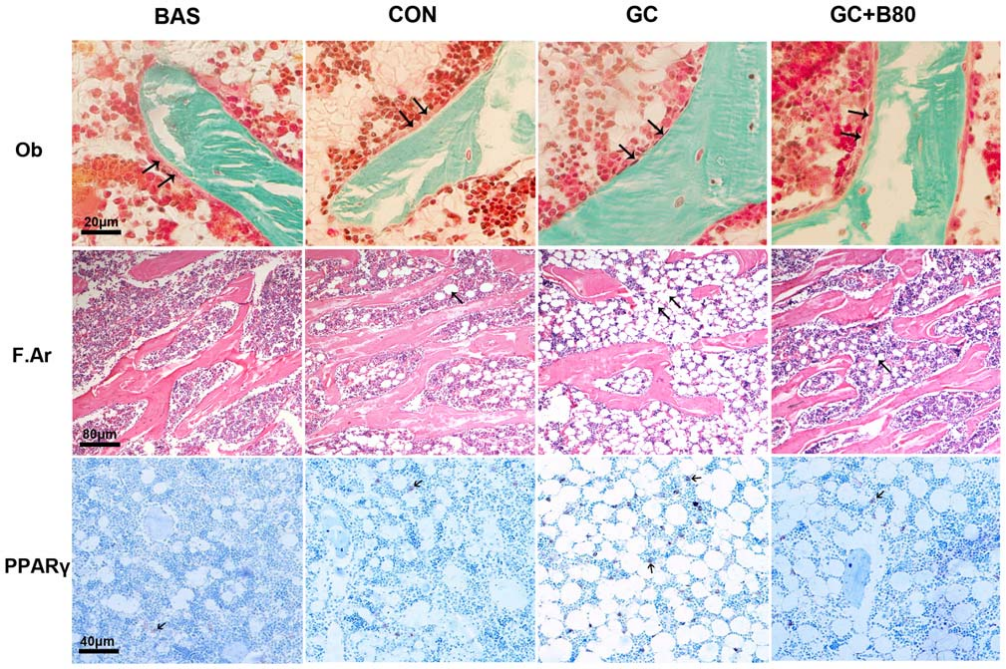
Effects of different treatments on osteoblast morphology and adipocyte distribution and corresponding PPARγ expression. Representative micrographs in basal (BAS), vehicle (aging) control (CON), prednisone (GC) and GC plus 80 mg Sal B/kg/d (GC+B80) treated cancellous bone in distal femoral metaphysic. Arrows point to osteoblasts (Ob, Goldner's Trichrome stain). Active osteoblasts are present as multi- plump columnar lining on the trabecular surface in BAS and CON rats. The GC treatment induced the appearance of shriveling and inactive osteoblasts (v.s. BAS & CON) while the Sal B treatment protected GC-induced osteoblast impairment (GC+B80 v.s.GC). Adipocyte content (F.Ar, Hematoxylin stain) and corresponding immunohistochemical staining of Peroxisome Proliferator-Activated Receptor γ (PPARγ) expression (arrows from spots, PPARγ stain) increased between 6 and 9 months (BAS v.s. CON). The GC treatment markedly increased adipocyte number and size (GC v.s. BAS & CON), and the amount of PPARγ positive cells, while the Sal B treatment prevented the GC-induced increases (GC+B80 v.s.GC). Quantitative measurements of osteoblasts, fatty area and PPARγ expression are shown in table 6 and table 8.

**Figure 9:**
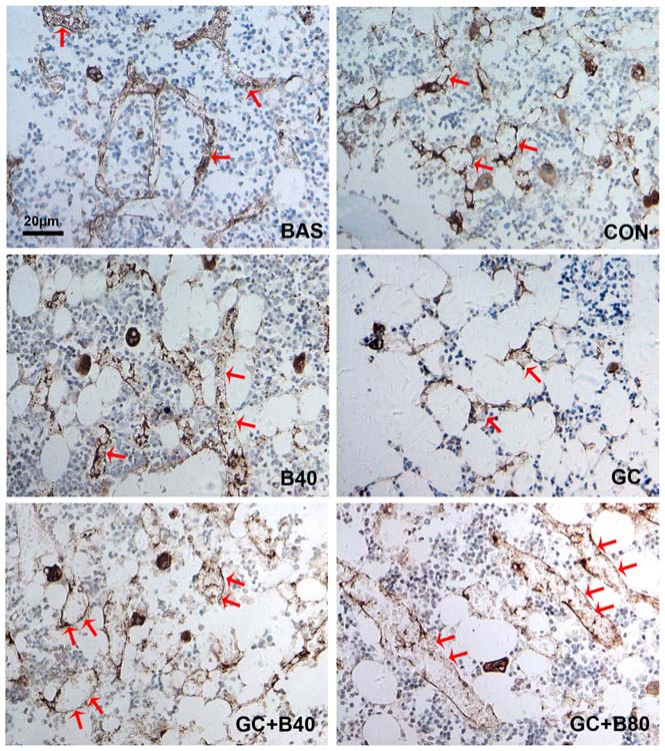
Effects of different treatments on marrow microvessel structures. Representative micrographs of immunohistochemical staining of Von Willbrand Factor (vWF) in microvessel endothelial cells of the distal femoral metaphysis from basal (BAS), aging control (CON), Sal B alone (B40), prednisone treated (GC), GC plus Sal B40 m g/kg/d (GC+B40) and GC plus Sal B80 mg/kg/d (GC+B80). The diameter of microvessels but not density increased between 6 and 9 months (BAS v.s. CON). The GC treatment markedly decreased the density and diameter of microvessels with the appearance microvessels squeezed by the increased adipocytes (GC v.s. BAS & CON). Sal B treatment prevented the GC induced changes. High dose (Sal B80 mg/kg/d) treatment significant increased the diameter of the microvessels (double arrows) (vs. GC & CON). Quantitative measurements of microvessel diameter (DMV) and density (MVD) are shown in table 8.

**Table 4:** Effects of Sal B, GC and GC+Sal B on the soft tissue weights (g/1000 g body weight).

Groups	Thymus	Liver	Adrenal gland (/100 g)	Testicles	Soleus
CON	0.53±0.06	26.45±1.28	0.90±0.10	4.17±0.29	0.46±0.04
B40	0.52±0.06	27.13±1.52	0.87±0.19	4.19±0.27	0.47±0.03
GC	0.40±0.07**	28.98±1.30**	0.72±0.19*	4.12±0.36	0.42±0.02*
GC+B40	0.48±0.08	28.33±2.86	0.89±0.12#	4.10±0.07	0.48±0.06#
GC+B80	0.43±0.09*	26.97±1.96#	0.90±0.17	4.11±0.26	0.47±0.04# #

Note: Value are mean ± SD,

* *P*<0.05,

** *P*<0.01 *vs* CON;

# *P*<0.05,

# # *P*<0.01 *vs* GC.

+*P*<0.05,

++*P*<0.01 *vs* GC+B40.

Not available for BSA.

**Table 5 pone-0034647-t005:** Effects of Sal B, GC, and GC+ Sal B on femur bone biomechanics.

Groups	Maximum force	Fracture Force	Elastic Force	Maximum	Stiffness coefficient
	(N)	(N)	(N)	deflection (mm)	(N/mm)
BAS	162.93±13.36**	134.58±22.16**	94.99±13.74**	0.97±0.19	531.09±49.67
CON	207.91±13.01	178.77±30.39	126.97±24.39	0.90±0.10	617.97±95.61
B40	208.52±21.50△△	171.37±38.07	121.35±38.26	0.90±0.17	678.24±51.53△△
GC	184.76±17.72* △	177.97±17.86△△	127.21±19.45△△	0.70±0.11* △	601.12±78.48
GC+B40	213.40±12.82# △△	191.98±21.22△△	131.75±10.85△△	0.94±0.14#	774.13±75.89# * △△
GC+B80	218.68±15.32# # △△	184.52±30.08△△	136.11±26.49△△	0.93±0.13#	738.87±57.53# * △△

Note: Value are mean ± SD,

* *P*<0.05,

** *P*<0.01 *vs* CON;

# *P*<0.05,

# # *P*<0.01 *vs* GC,

+*P*<0.05,

++*P*<0.01 *vs* GC+B40,

△ *P*<0.05,

△△ *P*<0.01 *vs* BAS.

**Table 6 pone-0034647-t006:** Effects of Sal B, GC, and GC+ Sal B on proximal tibial metaphysis bone structure histomorphometry, osteoblast and osteoclast contents.

Parameters	BAS	CON	B40	GC	GC+B40	GC+B80
BV/TV (%)	16.34±3.52	13.18±3.87	13.44±3.09	9.42±3.34* △△	14.04±2.56# #	17.58±3.20# # * +
Tb.Th (µm)	59.52±4.53	59.77±8.86	59.80±6.92	49.09±9.11** △	59.36±6.19#	69.82±8.85# # * + △
Tb.N (mm^−1^)	2.72±0.43	2.18±0.43	2.25±0.43	1.87±0.38△△	2.37±0.31#	2.51±0.25# #
Tb.Sp (µm)	316.2±68.6	416.3±103.1	399.02±93.3	505.3±123.5△△	369.51±51.3# #	332.4±47.7##
OcS/BS (%)	0.74±0.33	0.65±0.28	0.47±0.16△	0.86±0.36	0.79±0.31	0.55±0.30
ObS/BS (%)	1.21±0.33	1.21±0.42	1.50±0.68	0.31±0.13** △△	0.80±0.31#	1.01±0.36# #

Note: Value are mean ± SD,

* *P*<0.05,

** *P*<0.01 *vs* CON;

# *P*<0.05,

# # *P*<0.01 *vs* GC,

+ *P*<0.05,

++*P*<0.01 *vs* GC+B40,

△ *P*<0.05,

△△ *P*<0.01 *vs* BAS.

**Table 7 pone-0034647-t007:** Effects of Sal B, GC, and GC+ Sal B on proximal tibial metaphysis cancellou bone dynamic parameters.

Parameters	BAS	CON	B40	GC	GC+B40	GC+B80
LGR (µm/d)	8.31±0.45**	5.13±0.43	5.36±0.52△△	4.03±0.51** △△	5.10±0.25# # △△	5.32±0.42# # △△
MS (%)	23.11±2.92**	17.31±2.62	20.78±2.68* △	17.20±1.53△△	19.76±1.55# * △	18.40±0.99△
MAR (µm/d)	1.23±0.08	1.18±0.13	1.17±0.09	1.08±0.11* △△	1.21±0.07#	1.21±0.10#
BFR/BS (µm/d*100)	28.40±4.32*	20.49±3.93	24.40±4.08* △	18.68±3.41△△	23.95±2.86# # △	22.21±2.06△
BFR/BV (%/year)	291.5±43.7**	214.5±57.3	251.0±49.0	238.8±68.7△	247.1±32.3	198.8±22.2## + △
BFR/TV (%/year)	47.02±10.1**	26.53±4.13	32.87±4.94* △	21.19±4.97* △△	34.23±3.59# # * △	34.39±4.46# # ** △
Ec-MS (%)	84.95±10.7**	62.74±6.89	64.63±11.16△△	30.94±5.17** △△	50.95±9.55# # △△	59.08±9.88# # △△
Ec-MAR(µm/d)	3.55±0.75**	1.82±0.16	2.00±0.22△△	1.16±0.13** △△	1.59±0.25# △△	1.48±0.37△△
Ec-BFR/BS (µm/d*100)	298.7±61.3**	114.3±15.7	130.6±32.3△△	36.1±8.4** △△	79.5±11.3# # * △△	89.1±32.9# # △△

Note: Value are mean ± SD,

* *P*<0.05,

** *P*<0.01 *vs* CON;

# *P*<0.05,

# # *P*<0.01 *vs* GC,

+ *P*<0.05,

++*P*<0.01 *vs* GC+B40,

△ *P*<0.05,

△△ *P*<0.01 *vs* BAS.

**Table 8 pone-0034647-t008:** Effects of Sal B, GC, and GC+ Sal B on distal femoral metaphyseal bone marrow.

Parameters	BAS	CON	B40	GC	GC+B40	GC+B80
F.Ar/TV (%)	10.67±3.61	17.67±6.9	11.13±2.84	39.59±7.15** △	19.67±5.71# # △	21.23±4.53# △
PPARγ (A)	0.18±0.05# #	0.21±0.03	0.18±0.04	0.31±0.05** △△	0.23±0.03# △	0.20±0.03# #
DMV (µm)	9.74±2.92*	15.03±5.21	17.18±4.02△△	6.11±1.64** △	20.68±10.25# # △	21.23±6.86# # * △
MVD (vWF#/view)	24.7±1.64	26.1±1.61	27.0±1.56	20.1±3.68*	25.4±1.63#	27.9±2.57# # △

Note: Value are mean ± SD,

* *P*<0.05,

** *P*<0.01 *vs* CON;

# *P*<0.05,

# # *P*<0.01 *vs* GC,

+ *P*<0.05,

++ *P*<0.01 *vs* GC+B40,

△ *P*<0.05,

△△ *P*<0.01 *vs* BAS;

A: absorbance; vWF: positive staining of Von Willbrand Factor in the endothelial cells.

**Table 9:** Percentage change of all parameters from basal (BAS), vehicle control (CON) and predisone (GC).

%Change	Aging	B40		GC		B40+GC			B80+GC			
	v.s. BAS	v.s. BAS	vs. CON	v.s. BAS	v.s. CON	v.s. BAS	v.s. CON	v.s. GC	v.s. BAS	v.s. CON	v.s. GC	v.s. B40+GC
**BW**		12			−10						8	
**Thymus**	/	/		/	−25	/			/	−19		
**Liver**	/	/		/	10	/			/		−7	
**Adrenal Gland**	/	/		/	−20	/		24	/			
**Testicles**	/	/		/		/			/			
**Soleus**	/	/		/	−9	/		14	/		12	
**Calcium**	/	/		/	7	/		−5	/		−7	
**TRACP-5b**	/	/		/	42	/		−20	/		−20	
**ALP**	/	/		/	−52	/		46	/		104	40
**PF-BMD**				−22	−20			19			29	8
**Whole Femur BMD**		−8		−15	−11			22			10	
**Max force**	28	28		13	−11	31		16	34		18	
**Fracture Force**	33	(27)		32		43			37			
**Elastic Force**	34	(28)		34		39			43			
**Max Deflection**				−28	−22			34			33	
**Stiffness**		28				46	25	29	39	20	23	
**BV/TV**	(−19)			−42	−29			49		33	87	25
**Tb.Wi**				−18	−18			21	17	17	42	18
**Tb.N**	(−20)			−31				27		(15)	34	
**Tb.Sp**	(32)			60				−27		(−20)	−34	
**OcS/BS**	(−14)	−38	(−28)		(32)		(22)	(−8)		(−15)		
**ObS/BS**			(24)	−74	−74		(−34)	158		(−17)	226	
**LGR**	−38	−35		−52	−21	−39		27	−36		32	
**MS/BS**	−25	−10	20	−26		−14	14	15	−20			
**MAR**				−12	−8			12			12	
**BFR/BS**	−28	−14	19	−34	(−9)	−16		28	−22			
**BFR/BV**	−26			−18					−32		−17	−20
**BFR/TV**	−44	−30	24	−55	−20	−27	29	62	−27	30	62	
**Ec-MS/BS**	−26	−24		−64	−51	−40		65	−30		91	
**Ec-MAR**	−49	−44		−67	−36	−55		37	−58	(−19)	28	
**Ec-BFR/BS**	−62	−56	(14)	−88	−68	−73	−30	120	−70	(−22)	147	
**PPARy**	(17)		(−14)	72	48	28		−26			−35	
**Fat/TV**	(66)	(4)	(−37)	271	124	84		−50	99		−46	
**DMV**	54	76	(14)	−37	−59	112	(38)	238	118	41	247	
**MVD (vWF#/view)**					−23			26	13		39	

Note: This table shows significant %change from BAS, CON and GC, respectively. ( ): useful non-significant %changes. /: not available.

There were non-significant reductions in PTM cancellous bone mass (BV/TV down by 19%, Tb.N down by 20%, Oc.S/BS down by 14%), and increased Tb.Sp (up by 32%). In addition, PPARγ expression and marrow fatty area (F.Ar/TV) increased by 17% and 66% but did not reach significance. (Tables 6,8,9, Figures 7,8).

### Effect of Sal B40 in Intact Male Rats

Sal B40 treatment in intact male rats stimulated cancellous and endocortical bone formation. Compared to the vehicle (aging) control, the 40 mg of Sal B/kg/d (Sal B40) treated intact male rat showed no significant changes on body and soft tissues weights, bone mineral density (BMD), femoral cortical bone strength, PTM cancellous bone mass, architecture and endocortical bone formation. However, the Sal B40 treated rats showed significant increased select PTM cancellous bone formation indices (i.e. MS/BS, BFR/BS and BFR/TV up by 20, 19 and 24%), increase Ob.S/BS by 24% and Ec-BFR/BS by 14%. Additionally, the Sal B40 non-significantly reduced distal femoral marrow PPARγ expression by 14%, reduced marrow fatty area (F.Ar/TV) by 37% and increased DMV by 14%. (Tables 4–9; Figures 3–5,9)

### Effect of GC in Intact Male Rats

**Figure 10:**
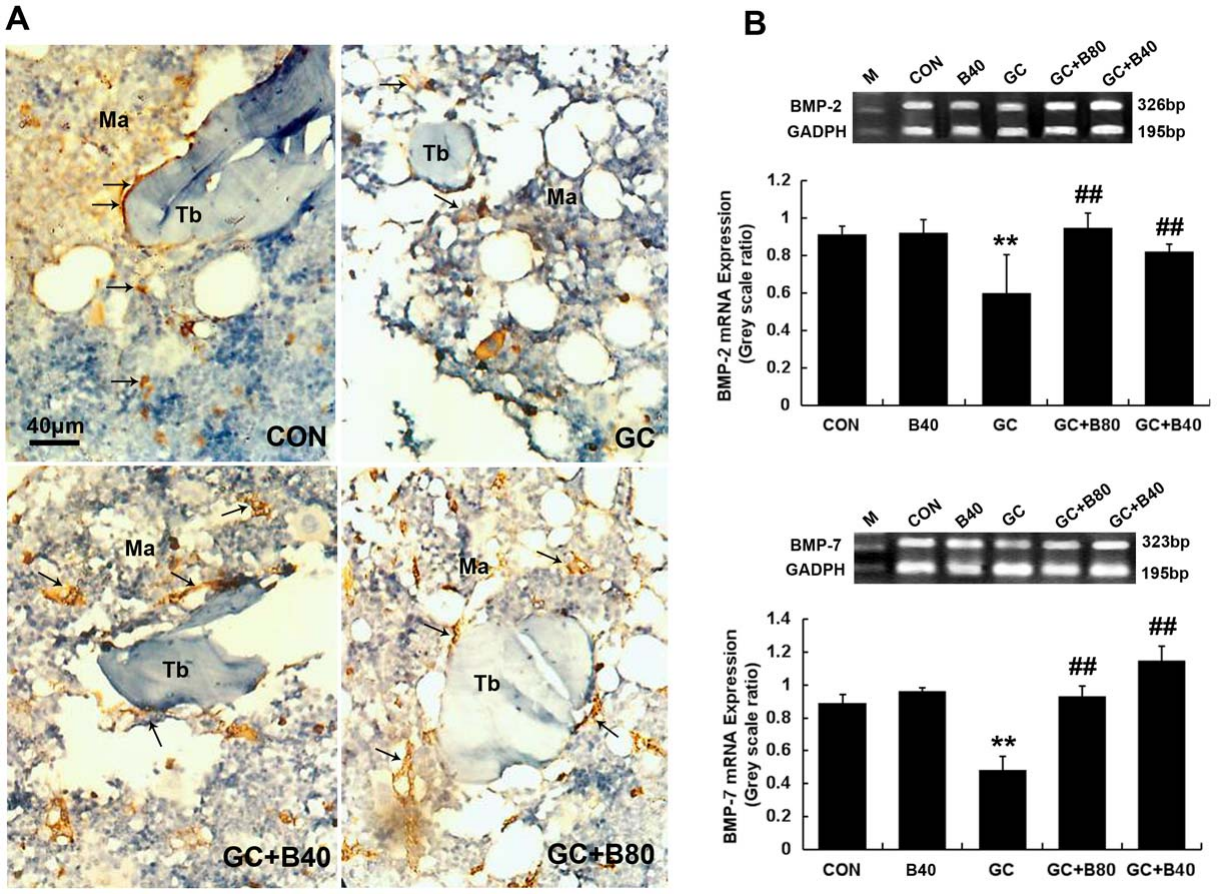
Effects of different treatments on BMP-2 and BMP-7 expression. A: Representative micrographs of immunohistochemical staining of BMP-2 in femur bone marrow and trabeculae from control (CON), prednisone treated (GC), GC plus Sal B40 m g/kg/d (GC+B40) and GC plus Sal B80 mg/kg/d (GC+B80). BMP-2 was stained as brown color with sand-like deposition along trabecular surface and marrow mesenchymal stem cells. The GC treatment markedly decreased the BMP-2 expression while Sal B treatment prevented the GC induced changes. B: Electrophoresis image of BMP-2 and BMP-7 mRNA expression in rat whole femur bone was determined by RT-PCR. The rats treated with GC decreased both BMP-2 and BMP-7 mRNA expression. Treatment of Sal 40 and 80 mg/kg/d completely prevented the GC induced changes, Sal 40 mg/kg/d alone did not affect the BMP-2 and BMP-7 mRNA expression. Value are mean ± SD, *P<0.05, ** *P*<0.01 *vs* CON; ^#^ *P*<0.05, ^# #^ *P*<0.01 *vs* GC.

GC induced losses in bone mass, BMD and mechanical strength associated with decreased osteogenesis, angiogenesis and increased adipogenesis. Compared to vehicle (aging) controls, the use of GC lead to significant increased of liver weight by 9.6%, and reduction in body, thymus, adrenal gland and soleus muscle weights by 10, 25, 20 and 9%, respectively, coupled with significantly increased serum calcium by 7%, tartrate-resistant acid phosphatase-5b (TRACP-5b) by 42% and decreased alkaline phosphatase (ALP) by 52%. Proximal femur and whole femur BMDs were decreased by 20% and 11% and cortical bone strength was reduced (i.e. maximum force and deflection by 11% and 22%). GC further significantly reduced PTM cancellous BV/TV by 29%, Tb.Wi by 18%, Ob.S/BS by 74%, PT LGR by 21%, and some PTM cancellous bone formation indices (i.e. MAR and BFR/TV) by 8 and 20%, Significant reductions were seen in the endocortical bone formation indices (i.e. Ec-MS/BS, Ec-MAR and Ec-BFR by 51, 36 and 68%), and microvessels diameter (DMV, down by 59%) and density (MVD) down by 23%, while fatty marrow content was increased by 124% and PPARγ expression by 48% (Table 4–9, Figure 6–9). Immunohistochemical analysis of bone marrow BMP-2 and BMP-7 expression significant decreased with decrease of total BMP-2 and BMP-7 mRNA expression (Figure 10).

### Effect of Sal B40 in GC treated Male Rats

Sal B40 treatment of GC-treated rats prevented most of the GC-induced changes. Compared to vehicle (aging) controls, the 40 mg Sal B/kg/d (Sal B40) - treated GC rats showed no significant differences in terms of body and soft tissues weights, serum bone biomarkers, BMD, biomechanical properties (except for increase in stiffness by 25%), PT LGR, PTM cancellous bone mass, architectures, MAR, BFR/BS, BFR/BV, Oc.S/BS and Ob.S/BS, endocortical formation indices (i.e. Ec-MS/BS, Ec-MAR, except for a decrease in Ec-BFR/BS by 30%), DMV and MVD, F.Ar/TV and distal femoral marrow PPARγ expression, and both mRNA and positive expression of BMP-2 and BMP-7. (Tables 4–9; Figure 3–5, 9–10)

Furthermore there were no differences in skeletal cell and tissues values, except for significantly increased PTM cancellous MS/BS (up by 14%) and BFR/TV (up by 29%), and decreased Ec-BFR/BS (down by 30%). (Tables 4–9; Figures 3–5)

The Sal B40 treated GC rats tended to exhibit a larger marrow DMV (up by 38%) versus aging control, that resulted in significantly increased DMV by 238%, when compared to GC control. The rats treated with Sal B40 showed reduced PTM cancellous bone Ob.S/BS by 34%, while the GC alone was reduced by 74%. The results suggests that both osteoblasts and microvessel diameters were stimulated by Sal B40. (Tables 4–9; Figures 3–5,9)

### Effect of Sal B80 in GC treated Male Rats

Sal B80 treatment of GC rats for 12 weeks not only prevented GC-induced changes but also showed additionally stimulated osteogenesis that increase cancellous bone mass and increased marrow angiogenesis by enlargement of microvessels diameter.

Compared to vehicle (aging) control, the GC treated rats receiving 80 mg of Sal B/kg/d (Sal B80) showed no significant differences in body and soft tissue weights, serum biomarkers, BMD, bone strength (except for stiffness), and marrow PPARγ expression, BMP-2 and BMP-7 expression, fatty marrow and MVD. However, the Sal B80 treated rats significant differed from Sal B40 treated rats in term of PTM cancellous bone mass up by 33%, Tb.Wi by 17%, BFR/TVby 30% and marrow DMV by 41% versus aging control. (Tables 4, 5, 8&9; Figures 3–9)

Compared to the Sal B40 treatment there was a significantly dose response increase in serum ALP level by 40%, in proximal femur BMD by 8%, in PTM cancellous BV/TV by 25%, in Tb.Wi by 18% and significantly decrease in BFR/BV by 20%. (Tables 4, 5, 8&9; Figures 3–9)

### Effect of Sal B in osteoblast viability and MSC differentiation *in vitro*

**Figure 11:**
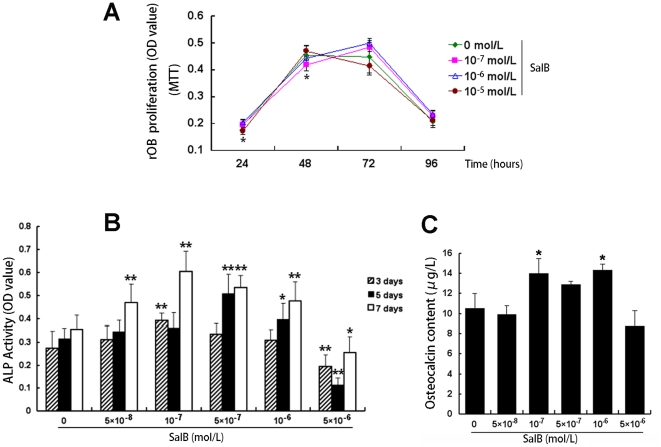
Effects of Sal B on the proliferation and differentiation in new born rat calvarium osteoblast. Cells were inoculated in 96-well plates and cultured, then transferred to a medium containing various concentrations of vehicle and Sal B. MTT was tested at 24, 48, 72 and 96 h of incubation (A), ALP activity (represent by OD value) was determined later until 7 days of incubation (B), and the content of osteocalcin (µg/L) in culture medium was determined 1n day 21 after incubated with different treatment (C). Data shown are mean ± SD. n = 6. **P<0.01, *P<0.01 versus control (vehicle treatment).

When cultured different concentration of Sal B with rOB, Sal B at concentration from 10^−8^ mol/L to 10^−6^ mol/L stimulated rOB cell growth and proliferation, increased ALP activity and osteocalcin secretion, this effect appeared in a time and dose-dependent manner, when the concentration reached 10^−5^ mol/L the cell death occurred with function decrease. (Figure 11).

**Figure 12:**
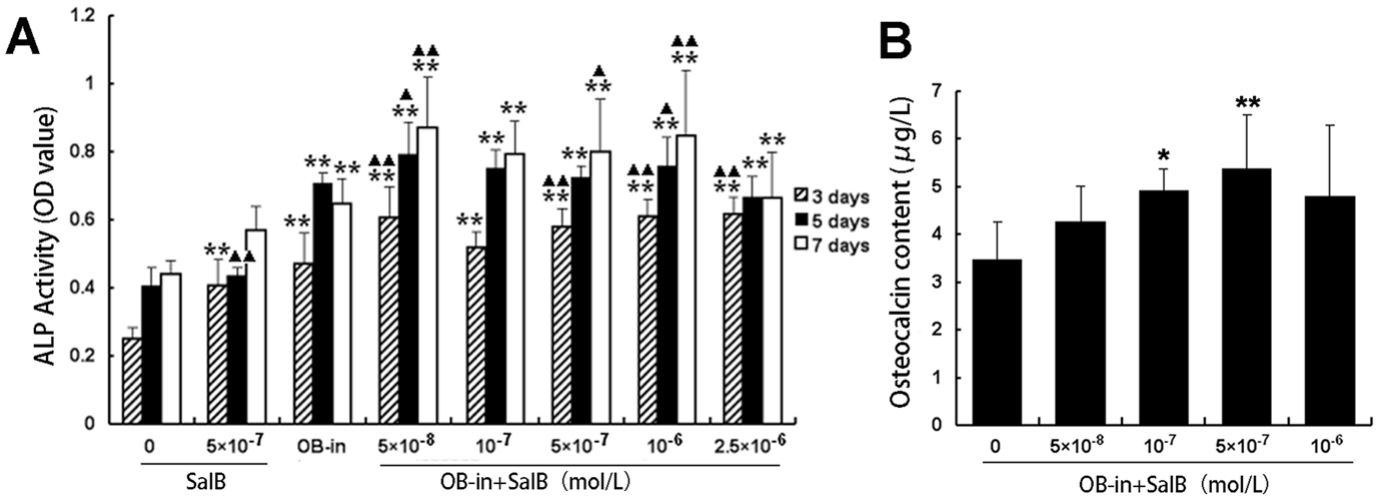
Effects of Sal B on rat marrow stromal cell (rMSC) differentiation into osteoblast. Cells were collected from the femur marrow of one month old SD rats. Cells inoculated in 25 mm^2^ culture flask and cultured. The cells used in the study were the 3 passage, then transferred to the medium containing osteoblast induction medium (OB-in), Sal B and OB-in plus various concentrations of Sal B respectively. A: ALP activity (represent by OD value) was determined at 3, 5 and 7 days. B: the content of osteocalcin (µg/L) in culture medium was determined 1n day 21 after incubated with different treatment. Data shown are mean ± SD. n = 6. **P<0.01, *P<0.05 versus control (vehicle treatment).

Sal B at 5×10^−7^ mol/L stimulated ALP secretion in MSC which was similar to the action of osteoblast induction media (OB-in), Sal B from 10^−8^ mol/L to 10^−6^ mol/L further increased OB-in treated-MSC secretion of ALP and osteocalcin, which reveal the ability of MSC differentiation into osteoblast by Sal B. (Figure 12)

### Effect of Sal B on Dickkopf-1(DKK-1)/β-catenin mRNA expression in MSC

**Figure 13:**
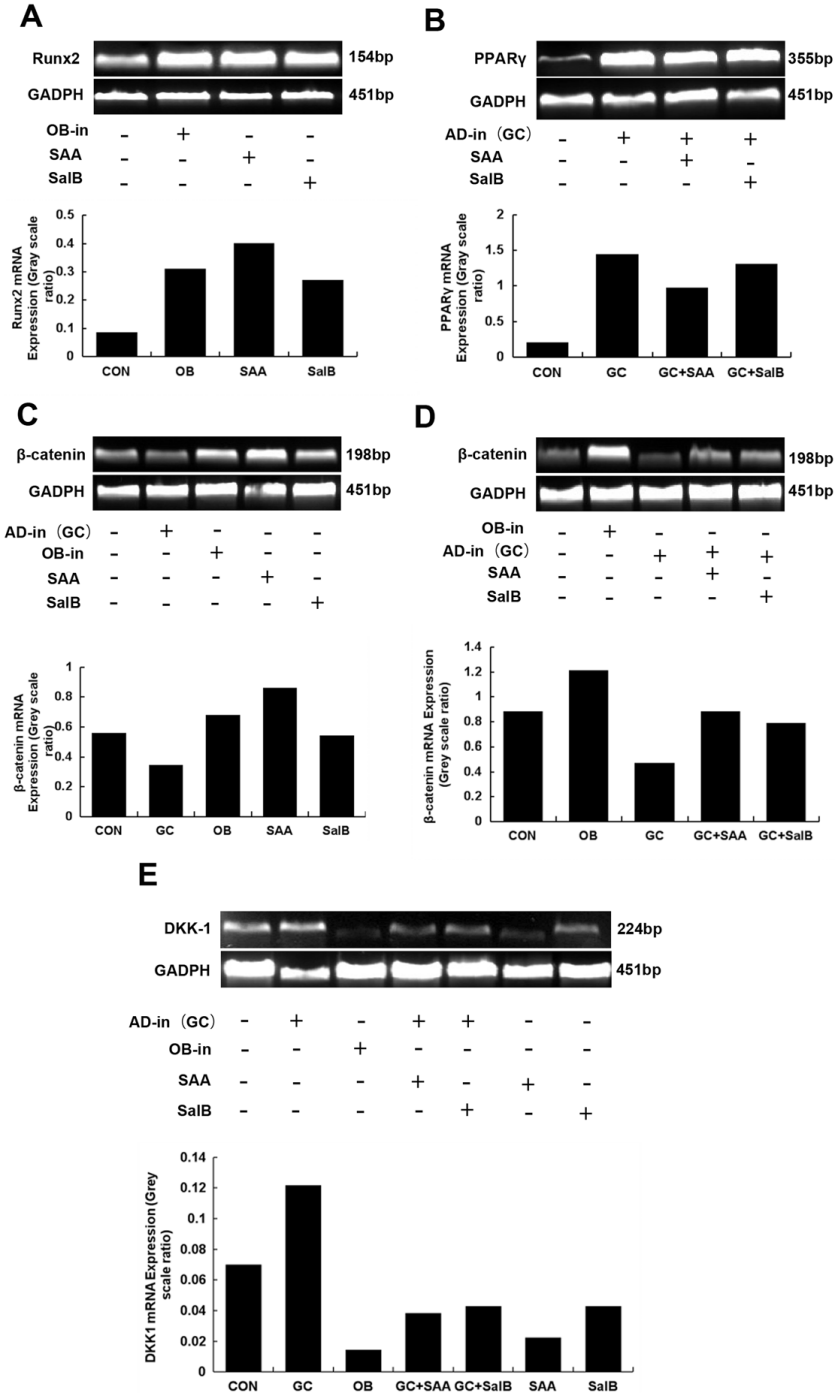
Effects of Sal B on the gene expression of Dkk-1/β-catenin pathway in rat marrow stromal cell (rMSC) differentiation. rMSC were cultured with osteoblast induction medium (OB-in), adipocyte induction medium (Ad-in, i.e. high concentration of GC), with or without Sal B (10-7 mol/L) and Sal A (5×10-7 mol/L, a derivant of Sal B, Figure 1) for 7 days. Expression levels of Runx2 (A) , PPARγ (B), β-catenin (C and D) and DKK-1 (E) were measured by RT-PCR. Sal B increased Runx2 and β-catenin mRNA expression and decreased DKK-1 mRNA expression which was similar to the action of OB-in. Ad-in marked increased PPARγ and DKK-1 mRNA expression. When treated the Ad-in rMSC with Sal B, the PPARγ and DKK-1 mRNA expression decreased obviously. Sal A had similar effects to Sal B.

DKK-1 is an inhibitor of Wnt signaling and GC is a strong stimulator of DKK-1. When MSCs were exposed to adipocyte induction medium (Ad-in, containing high concentration of GC), PPARγ mRNA expression increased accompanied with increases of DKK-1 mRNA expression while β-catenin mRNA expression decreased when compared to control. Treatment with 10^−7^ mol/L Sal B and 5×10^−7^ mol/L Sal A decreased PPARγ and DKK-1 mRNA expression and increased β-catenin mRNA expression with or without adipocyte inducement medium. Sal B 10^−7^ mol/L and Sal A 5×10^−7^ mol/L also increased Runx2 mRNA expression without osteoblast inducement medium (Figure 13).

## Discussion

The current study found that GC treatment decreased bone formation by inhibiting osteoblast activity and marrow production of BMP-2 and BMP-7, increased marrow adipocytes with elevated PPARγ expression that promoted bone marrow stromal cell differentiation into adipocytes. Since osteoblasts and adipocytes share common bone stromal progenitors [31], GC treatment stimulate the differentiation of marrow stromal cells to adipocytes thereby, reducing the pool of local progenitor cells to differentiate into osteoblasts, The reduction in osteoblast number in turn contributes to decreased osteogenesis. Furthermore, we specifically labeled marrow vascular endothelial cells with Von Willbrand Factor and observed similar GC disruption of marrow microvessels as previously observed in rabbit femoral head [32]. The disruption of marrow microvasculature would reduce the source of circulating progenitor cells supporting osteogenesis [33]–[39]. The reduction in local and circulating progenitors would then lead to reduce osteogenesis. Our findings suggest that bone formation, bone marrow fat metabolism and microcirculation are closely related to each other and confirm that the latter two factors also contribute significantly to the development of GC-induced bone loss and the decline in bone strength [4], [5].

Our data found 80 mg Sal B/kg/d for 12 weeks depressed adipogenesis and stimulated angiogenesis and osteogenesis in GC-treated rats. The lower 40 mg Sal B/kg/d dose in GC treated rats only depressed adipogenesis and stimulated cancellous bone formation rate. The 40 mg dose also appeared to increase angiogenesis by enlarging marrow microvessels diameter but the observations were not statistically significant. The prevention of GC-stimulation of adipogenesis maintains marrow stromal and red marrow cell levels at control levels. Since osteogenesis and adipogenesis share common bone stromal progenitors [31], this means the pool of marrow progenitors available to differentiate into osteoblasts will be maintained and not be preferentially stimulated to form adipocytes by GC treatment. Additionally the maintenance of red bone marrow means that the bone marrow will retain more microvessels, unlike fatty bone marrow, which is not as well vascularized [40], [41]. The Sal B treatment further increased marrow angiogenesis that increases blood flow to improve nutrition and source of circulating progenitors [33]–[39]. In support of osteogenesis, stimulated angiogenesis reduced intraosseous pressure that increases blood flow and in turn stimulates osteoblastic activity (i.e. mineral apposition rate) [42]. Further red bone marrow sites with better vasculature exhibit a factor of 10 higher bone remodeling and formation rates than fatty marrow bone sites [43]–[46]. Also the improved blood flow increases osteocyte lacunar-canaliculi and blood vessel fluid volume that increase bone strength and reduce the risk of fracture [4], [47].

To clarify the mechanism by which Sal B promotes osteoblast differentiation and alleviates GC-induced impairment of bone formation, we examined the effects of Sal B on primary rat calvarial osteoblasts and rat bone marrow stromal cell by up-regulating Wnt/β-catenin signaling. Our data showed that Sal B stimulates osteoblast cell growth and MSC differentiation into osteoblast maturation (secretion of ALP and osteocalcin) with a dose and time dependent manner that mimicks the action of osteoblast inducer. This study further demonstrated that Sal B activated Runx2 and β-catenin mRNA expression and declined DKK-1 and PPARγ mRNA expression which had been induced by GC (i.e. adipocyte inducement). It is known that canonical Wnt/β-catenin is a key pathway for regulating bone formation and remodeling and contributes to osteoblastic differentiation [48], of which DKK-1 is one of the inhibitor in Wnt-signaling. Studies demonstrate GC to be a very strong inducer of DKK-1 protein [49] which leads to a decrease in osteoblast bone formation in GC induced osteoporosis [50]. Moreover, Runx2 was found to integrate Wnt-signaling for mediating osteogenic differentiation of MSCs [51], it was known to be involved in BMP signaling as mentioned earlier [52]. Our in vivo observation further demonstrated that Sal B stimulated femur bone BMP-2 and BMP-7 mRNA expression and protected GC-treated rats from decrease of femur bone BMP-2 and BMP-7 protein expression (IHC revealed, Figure 10). BMP/Wnt/β-catenin share a crossover mechanism for stimulation of progenitor, osteoblast and angiogenetic generation [53], thus our study support the idea that Sal B protected against GC-induced osteoblast impairment associated abnormal angiogenesis and adipogenesis. Recently Lu et al. reported that Sal B protected against oxidative stress–induced apoptosis in rat bone marrow stem cells [54] suggesting that Sal B may inhibit GC-induced bone marrow stem cell apoptosis to enlarge the local pool of osteoblast precursors available for osteogenesis.

Our current end point findings indicated that Sal B stimulated bone formation in both intact and GC-treated male rats (Table 9). Sal B40 alone in intact male rats significantly increased BFR/TV by 24%, Sal B 40 in GC-treated rats significantly increased BFR/TV by 29%, while Sal B80 in GC-treated rats significantly increased BFR/TV by 50% and additionally increased cancellous bone mass (BV/TV) by 33% and trabecular thickness by 17%. Taken together, the combination of improved marrow microenvironment (i.e. increased local and circulatory progenitors, blood flow and nutrition) andSal B stimulation of bone formation rate not only prevented GC - induced osteopenia but also increased cancellous bone mass.

The current study used doses of 40 and 80 mg/kg gavage in rats, which is similar to the dose used in humans. Salvianolic Acid B (parental form) was approved by the State Food and Drug Administration (SFDA) of China in 2007 for clinical use in the prevention and treatment of cardiovascular diseases. The recommended dose of Salvianolic Acid B for humans is 4 mg/kg/d i.v. Our study examined the drug impact on body weight (Figure 3), organ weight (table 4), gross necropsy and histopathology (negative data not shown) both in intact rats (SalB40 group) and glucocorticoid-treated rats. No significant adverse effects on these parameters were observed. Recently Li et al. [55] reported on the toxicity of Sal A, a derivative of Sal B, in male and female dogs after a 3-month continuous intravenous infusion at doses of 17, 50, and 150 mg/kg/day. No significant cumulative toxicity was observed either during or 90 days following treatment. Importantly, the doses of Sal A used in dogs were very high as compared to clinical practice and to rats; the 17, 50, and 150 mg/kg/day in dogs are equivalent to 61.2, 180 and 540 mg/kg in rats. Sal B possesses a similar activity to Sal A. Our data indicate that the doses of Sal B used in current study are safe and effective and produce no adverse effects.

In summary, Sal B treatment of GC-treated male rats not only prevented GC-induced osteopenia but also increased cancellous bone mass by the combination of depressed adipogenesis and stimulated angiogenesis and osteogenesis (Figure S1). Our findings support further investigation of Sal B stimulating of osteogenesis and marrow circulation and inhibition of adipogenesis as a potential therapeutic strategy in the prevention of not only GC-induced osteopenia but other bone diseases.

## Supporting Information

Figure S1 **Working scheme: the mechanism of action of Salvianolic acid B on glucocorticoid induced bone loss.** Decrease of osteogenesis and angiogenesis, increase of adipogenesis are considered contributions to glucocorticoid induced bone loss. Salvianolic acid B could withstand the impairment induced by glucocorticoid. (BMPs: bone morphogenetic proteins; MSCs: marrow stromal cells; PPARγ: peroxisome proliferator-activated receptor γ; VEGF: vascular endothelial growth factor; Black: effect of glococorticoid; Red: effect of Salvianolic acid B; ↑: increase; ↓: decrease).
(DOC)

## References

[1] Shah SK, Gecys GT (2006). Prednisone-Induced Osteoporosis: An Overlooked and Undertreated Adverse Effect.. J Am Osteopath Assoc.

[2] Canalis E (2005). Mechanisms of glucocorticoid action in bone.. Curr Osteoporos Rep.

[3] Ito S, Suzuki N, Kato S (2007). Glucocorticoids induce the differentiation of a mesenchymal progenitor cell line, ROB-C26 into adipocytes and osteoblasts, but fail to induce terminal osteoblast differentiation.. Bone.

[4] Weinstein RS (2010). Glucocorticoids, osteocytes, and skeletal fragility: The role of bone vascularity.. Bone.

[5] Kerachian MA, Séguin C, Harvey EJ (2009). Glucocorticoids in osteonecrosis of the femoral head: a new understanding of the mechanisms of action.. J Steroid Biochem Mol Biol.

[6] Khan E, AbuAmer Y (2003). Activation of peroxisome proliferator 2 activated receptor 2 gamma inhibits differentiation of preosteoblasts.. J Lab Clin Med.

[7] Luppen CA, Chandler RL, Noh T, Mortlock DP, Frenkel B (2008). BMP-2 vs. BMP-4 expression and activity in glucocorticoid-arrested MC3T3-E1 osteoblasts: Smad signaling, not alkaline phosphatase activity, predicts rescue of mineralization.. Growth Factors.

[8] Yin L, Li YB, Wang YS (2006). Dexamethasone-induced adipogenesis in primary marrow stromal cell culture: mechanism of steroid-induced osteonecrosis.. Clin Med J (Engl.).

[9] Kitajima M, Shigematsu M, Ogawa K, Sugihara H, Hotokebuchi T (2007). Effects of glucocorticoid on adipocyte size in human bone marrow.. Med Mol Morphol.

[10] Miyanishi K, Yamamoto T, Irisa T, Yamashita A, Jingushi S (2002). Bone marrow fat cell enlargement and a rise in intraosseous pressure in steroid-treated rabbits with osteonecrosis.. Bone.

[11] Ersoy A, Kahvecioglu S, Ersoy C, Akdag I, Yurtsever I (2006). Is glucocorticoid-induced osteonecrosis after kidney transplantation related to osteoporosis?. Nephrol Dial Transplant.

[12] Marx N, Schonbeck U, Lazar MA (1998). PPAR2 gamma activators inhibit gene expression and migration in human vascular smooth muscle.. J Circ Res.

[13] Adams JD, Wang R, Yang J, Lien EJ (2006). Preclinical and clinical examinations of Salvia miltiorrhiza and its tanshinones in ischemic conditions.. Chin Med.

[14] Li YG, Song L, Liu M, Hu ZB, Wang ZT (2009). Advancement in analysis of Salviae miltiorrhizae Radix et Rhizoma (Danshen).. J Chromatogr A.

[15] Ji XY, Tan BKH, Zhu YZ (2000). Salvia miltiorrhiza and ischemic diseases.. Acta Pharmacol Sin.

[16] He HB, Yang XZ, Shi MQ, Zeng XW, Wu LM (2008). Comparison of cardioprotective effects of salvianolic acid B and benazepril on large myocardial infarction in rats.. Pharmacol Rep.

[17] Zhong J, Tang MK, Zhang Y, Xu QP, Zhang JT (2007). Effect of salvianolic acid B on neural cells damage and neurogenesis after brain ischemia-reperfusion in rats.. Acta Pharmaceut Sin.

[18] Lam FF, Yeung JH, Kwan YW, Chan KM, Or PM (2006). Salvianolic acid B, an aqueous component of danshen (Salvia miltiorrhiza), relaxes rat coronary artery by inhibition of calcium channels.. Eur J Pharmacol.

[19] Kang DG, Oh H, Chung HT, Lee HS (2003). Inhibition of angiotensin converting enzyme by lithospermic acid B isolated from radix Salviae miltiorrhiza Bunge.. Phytother Res.

[20] Zhou L, Zuo Z, Chow MS (2005). Danshen: an overview of its chemistry, pharmacology, pharmacokinetics, and clinical use.. J Clin Pharmacol.

[21] Cui L, Liu YY, Wu T, Ai CM, Chen HQ (2009). Osteogenic effects of D+beta-3,4-dihydroxyphenyl lactic acid (salvianic acid A, SAA) on osteoblasts and bone marrow stromal cells of intact and prednisone-treated rats.. Acta Pharmacol Sin.

[22] Chinese Pharmacopoeia Commission. (2005). Pharmacopoeia of People's Republic of China (Part I).

[23] Sun Y, Cui L, Wu T (2008). Preparation of Salvia miltiorrhiza utility aqueous extract by hydrochloric acid method and evaluation on osteoblast.. Chinese Pharmacological Bulletin.

[24] Wang H, Wang Q (2002). Quantitative determination of active constituents in complex prescription of salvia miltiorrhiza by HPLC.. Journal of China Pharmaceutical University.

[25] Padro T, Ruiz S, Bieker R (2000). Increased angiogenesis in the bone marrow of patients with acute myeloid leukemia.. Blood.

[26] Weidner N (1995). Current pathologic methods for measuring intratumoral microvessel density within breast carcinoma and other solid tumors.. Breast Cancer Res Treat.

[27] Baron R, Vignery A, Neff L, Silvergate A, Santa-Marria A, Recker RR (1983). Processing of undecalcified bone speciments for bone histomorphometry.. Bone histomorphometry: techniques and interpretation.

[28] Cui L, Wu T, Liu XQ, Li QN, Lin LS (2001). Preventive effects of ginsenoside on osteopenia of rats induced by ovariectomy.. Acta Pharmacol Sin.

[29] Jee WSS, Li XJ, Inoue J, Jee KW, Haba T, Takahashi H (1997). Histomorphometric assay of the growing long bone.. Handbook of Bone Morphology.

[30] Parfitt AM, Drezner MK, Glorieux FH (1987). Bone histomorphometry: standardization of nomenclature, symbols and units. Report of the ASBMR Histomorphometry Committee.. J Bone Miner Res.

[31] Blair HC, Sun L, Kohanski RA (2007). Balanced regulation of proliferation, growth, differentiation, and degradation in skeletal cells.. Ann N Y Acad Sci.

[32] Hu CG, Chen JC, Liu Q, He XJ (2004). Mechanism of femoral head necrosis induced by long term glucocorticoid treatment in rabbit model.. Chin J Orthop.

[33] Parfitt AM (1995). Problems in the application of in vitro systems to the study of human bone remodeling. Proceedings from the workshop on Human Models of Skeletal Aging. NIH.. Calcif Tissue Int.

[34] Parfitt AM (1998). Mini-review – Osteoclast precursors as leucocytes: Importance of the area code.. Bone.

[35] Brandi ML, Collin-Osdoby P (2006). Vascular biology and the skeleton.. J Bone Miner Res.

[36] Kuznetsov SA, Mankani MH, Cronthos S, Satomura K, Bianco P (2001). Circulation skeleton stem cells.. J Cell Biol.

[37] Hauge EM, Qvesel D, Eriksen EF, Mosekilde L, Melsen F (2001). Cancellous bone remodeling occurs in specialized compartments linked by cells expressing osteoblastic markers.. J Bone Miner Res.

[38] Eghbali-Fatourechi GZ, Lamsan J, Fraser D, Nagel D, Riggs BL (2005). Circulating osteoblast-lineage cells in humans.. N Engl J Med.

[39] Eriksen EF, Eghbali-Fatourechi GZ, Khosla S (2007). Remodeling and vascular spaces in bone.. J Bone Miner Res.

[40] Branemark PI (1958). Vital microscopy of bone marrow in rabbit.. Scand J Clin Lab Invest.

[41] Van Dyke D (1967). Similarity in distribution of skeletal blood flow and erythropoietic marrow.. Clin Orthop Relat Res.

[42] Reeve J, Arlot M, Wootton R, Edouard C, Tellez M, Hesp R (1988). Skeleton blood flow iliac histomorphometry and strontium kinetics in osteoporosis: a relationship between blood flow and corrected apposition rate.. J Clin Endocrinol Metab.

[43] Wronski TJ, Smith JM, Jee WSS (1980). The microdistribution and retention of injected 239Pu on trabecular bone surfaces of the beagle: implications for the induction of osteosarcoma.. Radiat Res.

[44] Wronski TJ, Smith JM, Jee WSS (1981). Variations in mineral apposition rate of trabecular bone within the beagle skeleton.. Calcif Tissue Int.

[45] Partfitt AM, Marcus R, Feldman D, Nelson DA, Prosen CJ (2008). Skeletal histo genelty and purpose of bone remodeling. Implication for the understanding of osteoporosis.. Osteoporosis. Third volume.

[46] Tian XY, Setterberg RB, Li X, Paszty C, Ke HZ, Jee WSS (2010). Treatment with a sclerostin antibody increases cancellous bone formation and bone mass regardless of marrow composition in adult female rats.. Bone.

[47] Liebschner MAK, Keller TS (2005). Hydraulic strengthening affects the stiffness and strength of cortical bone.. Ann Biomed Eng.

[48] Day TF, Guo X, Garrett-Beal L, Yang Y (2005). Wnt/beta-catenin signaling in mesenchymal progenitors controls osteoblast and chondrocyte differentiation during vertebrate skeletogenesis.. Dev Cell.

[49] Ohnaka K, Taniguchi H, kawate H (2004). Glucocorticoid enhances the expression of Dickkopf-1 in human osteoblast: novel mechanism of glucocorticoid -induced osteiporosis.. Biochem Biophys Res Commun.

[50] Ohnaka K (2006). Wnt signaling and glucocorticoid-induced osteoporosis.. Clin Calcium.

[51] Hamidouche Z, Hay E, Vaudin P, Charbord P, Schüle R (2008). FHL2 mediates dexamethasone-induced mesenchymal cell differentiation into osteoblasts by activating Wnt/beta-catenin signaling-dependent Runx2 expression.. FASEB J.

[52] Nakashima K, Zhou X, Kunkel G, Zhang Z, Deng J (2002). The novel zinc finger-containing transcription factor osterix is required for osteoblast differentiation and bone formation.. Cell.

[53] Das A, Botchwey E (2011). Evaluation of angiogenesis and osteogenesis.. Tissue Eng Part B Rev.

[54] Lu B, Ye Z, Deng Y, Wu H, Feng J (2010). MEK/ERK pathway mediates cytoprotection of salvianolic acid B against oxidative stress – induced apoptosis in rat bone marrow stem cells.. Cell Biol Int.

[55] Li G, Gao Y, Li S, Li C, Zhu X (2009). Study on toxicity of danshensu in beagle dogs after 3-month continuous intravenous infusion.. Toxicol Mech Methods.

