# Supplementary material for: Salvianolic Acid B Prevents Bone Loss in Prednisone-Treated Rats through Stimulation of Osteogenesis and Bone Marrow Angiogenesis
Source: PLoS One. 2012 Apr 6;7(4):e34647. doi: 10.1371/journal.pone.0034647 (PMC3321026; doi:10.1371/journal.pone.0034647)
Supplement: Figure S1 — Working scheme: the mechanism of action of Salvianolic acid B on glucocorticoid induced bone loss. Decrease of osteogenesis and angiogenesis, increase of adipogenesis are considered contributions to glucocorticoid induced bone loss. Salvianolic acid B could withstand the impairment induced by glucocorticoid. (BMPs: bone morphogenetic proteins; MSCs: marrow stromal cells; PPARγ: peroxisome proliferator-activated receptor γ; VEGF: vascular endothelial growth factor; Black: effect of glococorticoid; Red: effect of Salvianolic acid B; ↑: increase; ↓: decrease). (DOC) [file pone.0034647.s001.doc]

**Bone loss**

**Osteogenesis:** **↓**

BMPs↓Osteoblasts (ob)↓

Osteoprogenitors↓

MSCs differentiated to ob↓ Bone formation↓

**Angiogenesis:↓**

VEGF↓

Microvessel density↓

Diameter of Microvessel↓

Blood supply↓

**Adipogenesis:↑**

PPARγ ↑,

Marrow fat cells↑

Pressure of blood supply↑

**Prevention of**

**Bone loss**
